# Editorial Notes, News and Miscellany

**Published:** 1914-04

**Authors:** 


### Editorial Notes, News and Miscellany.

WHY DO intelligent, educated men say and write "on either side," when they mean, "on each side"?

THIRTY YEARS A SUBSCRIBER.—Dr. Daniel Parker of Calvert, Texas, renews his subscription to July 1, 1916, the beginning of Volume 31, and writes: "I have a stack of "The Red Backs" as high as my head, and every one a gem."

WANTED, a girl or woman in every town to take subscriptions for "The Red Back" on a fifty-cent commission. Canvass the women, to whom Mrs. Daniel's department every month contains matter of vital importance to wives and mothers.

DR. J. S. ABBOTT, Texas Commissioner of Pure Food, has resigned to accept a position in the inner circle of the bureau of chemistry in the United States Department of Agriculture. I understand that he and two other experts are to constitute a sort of a "clearing house," whatever that may be. Good luck to him. He is a very superior officer and will be a distinct loss to Texas.

THE CONFEDERATE VETERAN.—The death of Mr. Cunningham, the founder of *The Veteran,* will cause no interruption in its publication. Mr. Cunningham "willed it to the people," and appointed a board of trust to have charge of the business. Every family in the South should subscribe for *The Confederate Veteran.* Send postal for sample copy. Subscription, $1.00 per year. *The Veteran* is of age—twenty-one years old—and has had a larger circulation for a longer time than any monthly ever had in the South.

---

THE WONDERFUL LYDSTON.—Elsewhere in this issue we reproduce from the *Bulletin of the Chicago Medical Society* a brief account of the successful transplanting *to his own person* of a testis from a man who had been dead twenty-four hours, and also the transplanting of an ovary from a woman who had been dead twenty-three hours, to an old broken down woman. It is simply wonderful—amazing. Dr. Carrell will have to look to his laurels. Dr. Lydston will make a full and detailed report of the case and it will appear in "The Red Back."

---

BOARDS of commissioned medical officers will be convened to meet at the Bureau of Public Health Service, 3 B Street, S. E., Washington, D. C., and at the marine hospitals of Boston, Mass.; Stapleton, N. Y.; Chicago, Ill.; St. Louis, Mo.; New Orleans, La., and San Francisco, Cal., on Monday, April 27, 1914, at 10 o'clock a. m., for the purpose of examining candidates for admission to the grade of assistant surgeon of the Public Health Service, when applications for examination at these stations are received in the bureau. Write to the surgeon general for details.

---

NASAL AND AURAL INFECTIONS.—An open letter to the profession which is appearing in leading medical journals over the signature of Parke, Davis & Co., adduce additional evidence of the value of Mixed Infection Phylacogen in stubborn nasal and aural infections. This communication, which bears the title, "A Letter to Medical Men," cites some cases that appear strongly confirmatory of the mixed-infection theory of etiology. All of the reports are interesting. At least one of them is remarkable; it deals with a housemaid who suffered almost total deafness in one ear for twenty-one years and whose hearing in the defective organ was practically restored after eleven injections of Mixed Infection Phylacogen.

---

THE AMERICAN SOCIETY FOR THE CONTROL OF CANCER has made arrangements for a special meeting on cancer to be held at the New York Academy of Medicine, 17 West Forty-third Street, on Friday evening, April 10, 1914, at 8:30 p. m. This date has been selected in order that physicians and surgeons attending the Congress of the American Surgical Association may conveniently be present. The public is also invited.

Dr. William J. Mayo, of Rochester, Minn., president of the American Surgical Association, and Frederick L. Hoffman, LL. D., statistician of the Prudential Insurance Company, will discuss the problem of cancer and its control. Dr. Francis Carter Wood, director of the George Crocker Research Laboratory, will describe the investigation of cancer which has been undertaken there under the auspices of Columbia University. Dr. J. Collins Warren of Boston, professor emeritus of Harvard University, and president of the Harvard Cancer Commission, will give an account of the establishment of that commission and the research work which it is directing at the Collis P. Huntington Memorial Hospital, and the Harvard Medical School.

---

The Harrison Anti-Narcotic Bill now before Congress, it seems to us, discriminates unjustly against heroin as to morphine, the respective amounts allowed per ounce under the "Exceptions" being a fourth grain of morphine, and only one-twelfth grain of the weaker drug, heroin. This is unjust to the physician, the manufacturer, and the retail druggist; the proportions should be reversed. My attention has been called also to an objectionable feature which it seems will be especially hard on the physician practicing in the country. The bill does not allow a physician to supply himself with such products as morphine or cocaine as required in his office practice or in his grip in making visits, for immediate use in treating those requiring his services, without first registering with the internal revenue department and supplying himself with order blanks, as furnished to other individuals for the purpose of purchasing such products. For example: As in the case of physicians practicing in rural districts, it would be difficult for them to comply with the law in this respect, and this would often result in much suffering and danger to those depending upon his services. It seems to us that the manufacturer should be given a little more leeway in supplying the registered physician with what he may require in such instances. Physicians should write to their Congressmen.

---

Significance of Earache.—Irving Sobotky pleads with the practitioner to treat earache with the respect it deserves, and emphasizes these points: (1) The cause of the earache must be determined; (2) treatment must be prompt; (3) the patient must be closely watched for complications (mastoiditis); (4) if complications arise, despite careful treatment, the patient must be immediately referred to an otologist; (5) in repeated attacks of earache, nasal irrigations and violent blowing of the nose must be prohibited; (6) enlarged tonsils and all adenoid tissue must be removed.

